# CALCA and TRPV1 genes polymorphisms are related to a good outcome in female chronic migraine patients treated with OnabotulinumtoxinA

**DOI:** 10.1186/s10194-019-0989-9

**Published:** 2019-04-23

**Authors:** R. Moreno-Mayordomo, M. Ruiz, J. Pascual, M. Gallego de la Sacristana, I. Vidriales, M. Sobrado, E. Cernuda-Morollon, A. B. Gago-Veiga, D. Garcia-Azorin, J. J. Telleria, A. L. Guerrero

**Affiliations:** 1 Clinical Analysis Department, Hospital Clínico Universitario de Valladolid, Valladolid, Spain; 2 Headache Unit, Neurology Department, Hospital Clínico Universitario de Valladolid, Avda. Ramón y Cajal 3, 47005 Valladolid, Spain; 3 Neurosciences Department, Hospital Universitario Central de Asturias, Oviedo, Spain; 4 Hospital Universitario Marqués de Valdecilla, Santander, Spain; 5 Neurology Department. Hospital Universitario de La Princesa, Madrid, Spain; 6 Instituto de Biología y Genética Molecular, University of Valladolid, Valladolid, Spain; 7 Instituto de Investigación Biomédica de Salamanca (IBSAL), Salamanca, Spain

**Keywords:** CALCA gene, Chronic migraine, OnabotulinumtoxinA, Single nucleotide polymorphisms, TRPV1 gene

## Abstract

**Background:**

Some variables have been proposed as predictors of efficacy of OnabotulinumtoxinA in chronic migraine patients, but data available are inconclusive. We aimed to analyse the influence of single nucleotide polymorphisms in the response to OnabotulinumtoxinA.

**Methods:**

We included 156 female patients treated with OnabotulinumtoxinA accordingly to PREEMPT paradigm in three headache units. OnabotulinumtoxinA was offered to patients that had not responded to topiramate and at least one other preventative. Age at first procedure was 43.7 ± 11.8 years (16–74). Patients with a reduction of at least 50% in the number of migraine days after two OnabotulinumtoxinA procedures were considered as responders. We analysed 25 polymorphisms selected for their relevance regarding migraine pathophysiology and their association with migraine according to previously published genome-wide association studies. Genotyping was performed using KASP probes and a LightCycler-480 (Roche-Diagnostics). Allelic, genotypic frequencies and dominance/recesivity hypothesis of the allelic variants were compared between responders and non-responders by Fisher’s exact test.

**Results:**

Response to treatment with OnabotulinumtoxinA was achieved in 120 patients (76,9%). Two polymorphisms showed differences: CALCA rs3781719, where allele C represents 26.9% in responders and 40.9% in non-responders (*p* = 0.007, OR = 3.11 (1.33–7.26)); and TRPV1 rs222749, where allele A represents 4.17% in responders and 12.5% in non-responders (*p* = 0.013, OR = 3.29 (1.28–8.43)). No significant differences in rest of polymorphisms or clinical or demographic variables were found.

**Conclusions:**

Polymorphic variations of CALCA and TRPV1 genes might play a role as prognostic markers of efficacy of OnabotulinumtoxinA in chronic migraine female patients in our population.

## Background

Chronic migraine (CM) is a debilitating neurological disorder which affects approximately 2–3% of adults and is five times more frequent among female. The pathophysiology of migraine attacks is based on the activation of the trigeminovascular system and the liberation of vasoactive neuropeptides which cause meningeal inflammation and produce pain, such as the calcitonin gene related peptide (CGRP) [1].

OnabotulinumtoxinA (OnabotA) is considered a safe and effective therapy to manage CM, as was shown in the PREEMPT clinical program [2–5]. Also in a real-life clinical practice OnabotA pericranial injections effectively reduced headache and migraine days [5–11].

Molecular mechanism of action of OnabotA consists on cleaving SNAP-25 and, so, impairing intracellular vesicular fusion and down regulating release of pain related neuropeptides, such as glutamate, substance P or CGRP [12, 13]. In addition, OnabotA blocks the translocation of membrane receptors to the surface of sensory neurons, such as the Transient Receptor Potential Vanilloid 1 (TRPV1) [14]. Decreasing peripheral sensitization by all these mechanisms, OnabotA would finally indirectly inhibit central sensitization [15].

Genetics is considered a key factor for migraine susceptibility. Some genes have been postulated as responsible for the appearance of migraine. These are related to ion channel and neurotransmitter pathways (glutamate, GABA, dopamine, serotonin) [16], vascular functions (CGRP, MTHFR) [16, 17], hormonal mechanisms [16] and nociceptive system (like receptors of the transient receptor potential family or TRP) [18].

Up to date, many predictors of response to OnabotA in CM patients have been proposed. They have been mainly related with the mechanism of action of OnabotA (as plasma levels of CGRP) [19] and with parameters that might imply a loss of possibility of dechronification of migraine as age, time from onset of migraine or chronic migraine [20], or structural or functional changes in pain related brain structures [21]. Data regarding a possible influence of migraine characteristics (strictly unilateral location, implosive pain, the presence of cutaneous allodynia or pericranial tenderness) have shown inconclusive [22].

We aimed to analyse single nucleotide polymorphisms (SNPs) previously related with a possible susceptibility to migraine, and to determine their value as prognostic markers of efficacy, in a population of CM patients treated with OnabotA.

## Methods

### Patients and study design

A prospective, observational, multicentre study was conducted in which demographic and clinical data, along with biological samples, were collected from 156 female patients affected by CM between January 2014 and December 2015.

20 male patients were firstly included in the cohort but not analysed as we found this number too low for comparison.

The patients were recruited in the Headache Units of three Tertiary Hospitals: *Hospital Clínico Universitario de Valladolid*, *Hospital Central de Asturias* and *Hospital Universitario de La Princesa*. All of them were of Caucasian ethnicity and Spanish origin, and no siblings were included.

Written informed consent was obtained from all the patients to perform genetic analysis.

Scientific as well as ethical approvals were obtained from the Clinical Research Ethics Committee (CEIC) of East Valladolid Area (Hospital Clínico Universitario de Valladolid) as well as CEIC of Hospital La Princesa, according to the Declaration of Helsinki (1975). Patient anonymity and adherence to Data Protection Laws were maintained at all times.

CM was diagnosed accordingly to International Classification of Headache Disorders, III edition, beta version [23]. All patients included were eligible for treatment with OnabotA in accordance with the PREEMPT protocol. Exclusion criteria for the use of OnabotA were pregnancy, breastfeeding, and drug or alcohol abuse. We did not exclude medication overuse and our patients were allowed to continue with previous preventatives with no dose increasing.

License of OnabotA in Spain indicates its use “for patients who have not adequately responded or are intolerant to prophylactic drugs for migraine”. Inclusion criteria in our study considered patients previously treated with topiramate (or another neuromodulator in case of intolerance) and at least one other preventative (among beta-blockers or calcium antagonists) with no efficacy. Lack of response was evaluated after the administration of these drugs at adequate doses during at least 3 months, unless intolerance.

OnabotA was injected accordingly to PREEMPT protocol. The patients used a diary in order to record migraine and headache days and the days in which symptomatic medications were used. We also gathered time (years) from onset of migraines and time (months) from onset of chronic migraine. Response to treatment with OnabotA was evaluated 3 months after the second procedure and was defined as a reduction of at least 50% in the number of monthly migraine days.

We analysed 25 SNPs from each patient; they were selected for their relevance regarding migraine pathophysiology and their association with migraine according to previously published genome-wide association studies (GWAS). So, some polymorphisms were directly related to an increasing in the occurrence of migraine (MEF2D, MTHFR, MTDH, TRPV1 GABRQ, GABRE and GABRA3, this latter only in the case of female population), and others with processes that might be part of it: SLC1A2 were linked to the tendency to excessive use of symptomatic medication in migraine patients, GRIK3 with schizophrenia, which shares with migraine processes related to the glutamatergic system, and SCN9A, P2RX7 and KCNS1 with increased pain chronification [24–31].

Genes and SNPs studied as well as the change in DNA sequence (NM or NC), changes caused to proteins (NP), its OMIM reference number and the position of each gene in each chromosome are shown in Table 1.

**Table 1:** Genes and SNPs studied

GEN	SNP	NM	NP	OMIM	POSITION
Genes related to glutamate homeostasis					
MEF2D	rs1050316	NM_001271629.1:c.*2734C>A	–	608320	15q26.3
LRP1	rs11172113	NM_002332.2:c.67+4469T>C	–	190182	3p24.1
MTDH	rs1835740	NC_000008.10:g.98166913 T > C	–	610323	8q22.1
EAAT2	rs4354668	NM_001195728.2:c.-130+507A>C	–	600300	11p13
GRIK3	rs6691840	NM_000831.3:c.928T>C	NP_000822.2:p.Ser310Ala	138243	1p34.3
Gene which encodes for CGRP					
CALCA	rs3781719	NC_000021.8:g.22052312C>T	–	114130	11p15.2
	rs145837941	NM_001033952.2:c.197 T > C	NP_001029124.1:p.Leu66Pro		
Genes of the GABA system					
GABRE	rs1139916	NM_004961.3:c.304T>G	NP_004952.2:p.Ser102Ala	300093	Xq28
GABRQ	rs3810651	NM_018558.3:c.1432A	NP_061028.3:p.Ile478Phe	300349	
GABRA3	rs6627221	NM_000808.3:c.551+8376A>G	–	305660	
	rs2201169	NM_000808.3:c.262+23738T>C	–		
Genes which encode voltage dependant channels					
SCN9A	rs6746030	NM_002977.2:c.3448C	NP_002968.1:p.Arg1150,	603415	2q24.3
KCNS1	rs734784	NM_002251.3:c.1465A>G	NP_002242.2:p.Ile489Val	602905	20q13.12
P2RX7	rs2230912	NM_002562.5:c.1379A>G	NP_002553.3:p.Gln460Arg	602566	12q24.31
	rs1718119	NM_002562.5:c.1042G>A	NP_002553.3:p.Ala348Thr		
Gene which encodes the D2 dopamine receptor					
DRD2(ANKK1)	rs1800497	NM_178510.1:c.2137G>A	NP_848605.1:p.Glu713Lys	608,774	11q23.2
Gene of the 5-HT2C serotonin receptor					
HTR2C	rs3813929	NM_000868.2:c.-759C>T	**–**	312861	Xq23
Genes of the TRP family					
TRPV1	rs222749	NM_018727.5:c.271C>T	NP_061197.4:p.Pro91Ser	602076	17p13.2
	rs222747	NM_018727.5:c.945G>C	NP_061197.4:p.Met315Ile		
	rs222741	NM_080704.3:c.-34+2841C>T	–		
TRPV3	rs7217270	NM_001258205.1:c.2085+395T>C	–	607066	17p13.2
TRPM8	rs10166942	NM_024080.4:c.-990T>C	–	606678	2q37.1
Other genes studied					
WFS1	rs734312	NM_001145853.1:c.1832G>A	NP_001139325.1:p.Arg611His	606201	4p16.1
TGFBR2	rs7640543	NC_000003.11:g.30462403G > A	–	190182	3p24.1
MTHFR	rs121434294	NM_005957.4:c.547C > T	NP_005948.3:p.Arg183Ter	607,093	1p36.3

Considering that this was the first study addressing the relationship between the variants and genes and OnabotA response, we estimated the sample size using the most similar study from a similar population [28]. We evaluated the highest possible variance (0.164) and the smallest possible difference in the proportions (0.15), with a type 1 error of 5% and a statistical power of 90%, with a possible attrition rate of 15%, with a minimum estimated sample size of 147 patients.

### Sample collection and genotyping

Three mL of peripheral blood anticoagulated in EDTA-K_3_ were collected from each patient and from this was extracted the genomic DNA using the commercial kit “Ultra Clean® Blood DNA Isolation”, following the manufacturer’s protocol. The DNA obtained was quantified using the spectrophotometer NanoDrop ND1000 (Thermo Fisher Scientific, Inc.®) and genotyping was then carried out with KASP probes (*Kompetitive Allele Specific PCR*, KBioscience®) which are based on polymerase chain reaction (PCR) with the simultaneous use of two specific forward primers, each one of which differs in only one nucleotide so as to be able to recognise the two possibilities of alleles which exist in a point mutation or SNP.

The fluorescent emissions of the PCR product were measured at 37 °C in a LightCycler 480® (Roche Diagnostics). The absorbance measure determined the alleles (in homo or heterozygosis) according to the fluorescence emitted by the fluorophores FAM and/or HEX. The homozygotes emitted the signal of only one fluorophore and the heterozygotes of both.

### Statistical analysis

Using the Fisher’s exact test, an assessment was made to see if the genotypic frequencies of each variant fulfilled the Hardy-Weinberg equilibrium.

Subgroups were established within the cohort of patients: responders and non-responders. We firstly analysed if clinical and demographic variables related with the response to OnabotA. We also searched for any differences between the groups in relation to any of the 25 SNPs for allelic, genotypic frequencies and dominance/recesivity hypothesis of the allelic variants. For this purpose, 3 × 2 contingency tables were used to analyse genotypic distribution and 2 × 2 contingency tables for the analysis of allele distribution and of allele dominance/recesivity, using the Fisher’s exact test.

The statistical analysis was carried out using version 20.0 of the IBM-SPSS programme for Windows. Values for *p* of less than 0.05 were considered to be statistically significant, taking into account the Bonferroni correction.

In all cases where a significant association was found, a binary logistic regression was performed, adjusting for age, in order to determine the possible influence of this variable in the signification found.

To estimate the risk derived from the significant differences, the *Odds Ratio* (OR) statistical measure was calculated with a 95% confidence interval. Thus, several comparisons were made for those which obtained a value of the odds ratio: for the allele of greater risk versus the allele of less risk and for risk according to three genetic models: dominant, codominant and recessive, using Bonferroni adjustment for multiplicity correction.

## Results

Among the 156 patients included in the study, 91 (58.3%) were included in Valladolid, 10 (6.4%) in Madrid and 55 (35.3%) in Asturias. Age at inclusion was 43.7 ± 11.8 years.

Response to treatment with OnabotA was achieved in 120 patients (76.9%).

None of the demographic variables considered predict significantly response to OnabotA.

All of the variants analysed were found to be in Hardy-Weinberg Equilibrium except one. This exception was SNP rs222749, located on the gene TRPV1 (*p =* 0.00006).

Results regarding relationship between SNPs and response to OnabotA are shown in Appendices 1, 2.

Among the variables studied, after correction for multiple comparisons, there were two which showed significant differences between the groups of responders and non-responders.

Significant differences in SNP rs3781719 of the gene CALCA were found. Allele C represented 40.9% of non-responders and only 26.9% of responders (Table 2). For this SNP, the results of genotype determination in 7 samples were inconclusive, so the cohort consisted of 149 patients instead of 156.

**Table 2:** Association between response to OnabotA and SNP rs3781719 in the gene CALCA

						*P value*				*Odds ratio* (95% CI)		
POPULATION	RESPONSE	GENOTYPES			MINOR ALLELE FREQUENCY (%)	GENOTYPIC FREQUENCY	ALLELE FREQUENCY	ALLELE C RECESIVITY HYPOTHESIS	ALLELE C DOMINANCE HYPOTHESIS	Dominant model	Codominant model	Recessive model
		TT	TC	CC								
*n* = 150	Responders (*n* = 117)	63	45	9	26,9	0.023	0.029	0.794	0.007	3,11 (1,33-7,26)	1,6 (0,85-3,0)	1,2 (0,31-4,71)
	Non-responders (*n* = 33)	9	21	3	40,9							

Regarding the strength of association, the following values of *Odds Ratio* were obtained with confidence intervals of 95%: allele C related with a greater risk of non-response than allele T: [OR = 1.88 (1.06–3.32)] and, in relation to genetic models, the dominant model had a value of OR = 3.11 (1.33–7.26); the codominant model had a value of OR = 1.6 (0.85–3.00) and the recessive model had a value of OR = 1.2 (0.31–4.71).

The SNP rs222749 located on the gene TRPV1 also showed significant differences between the two groups. Allele A represented 12.5% in non-responders and 4.17% in the group of responders (Table 3).

**Table 3:** Association between response to OnabotA and SNP rs222749 in gene TRPV1

						*P value*				*Odds ratio* (95% CI)		
POPULATION	RESPONSE	GENOTYPES			MINOR ALLELE FREQUENCY (%)	GENOTYPIC FREQUENCY	ALLELE FREQUENCY	ALLELE A RECESIVITY HYPOTHESIS	ALLELE A DOMINANCE HYPOTHESIS	Dominant model	Codominant model	Recessive model
		GG	GA	AA								
*n* = 156	Responders (*n* = 120)	111	8	1	4,2	0,040	0,021	0,013	0,102	2,47 (0,81-7,48)	2,40 (0,80-7,24)	10,82 (1,09-107,45)
	Non-responders (*n* = 36)	30	3	3	12,5							

Regarding the strength of association, the following values of *Odds Ratio* were obtained with confidence intervals of 95%: allele A related with a greater risk of non-response than allele G [OR = 3.29 (1.28–8.43)] and, in relation to genetic models, the dominant model had a value of OR = 2.47 (0.81–7.48); the codominant model had a value of OR = 2.40 (0.80–7.24) and the recessive model had a value of OR = 10.82 (1.09–107.45).

All the results were confirmed by age-adjusted logistic regression.

None of the clinical and demographic variables considered in our population predicted OnabotA response.

## Discussion

Efficacy of OnabotA in CM patients in our series is comparable to recent “real-life” data [10].

There are lots of SNPs that have been associated with migraine in GWAS [24–31], and have been validated with other independent studies. These SNPs are located in genes related to migraine pathophysiology. So occurs with glutamate, the main excitatory neurotransmitter in the central nervous system (CNS) whose levels have been shown increased in the cerebrospinal fluid and serum of migraineurs. Another example is Gamma-Amino Butyric acid (GABA), the main inhibitory neurotransmitter in the CNS. Also with CGRP, a powerful vasodilator neuropeptide and mediator of neurogenic inflammation. Or with the methylene-tetrahydrofolate reductase (MTHFR) system and the angiotensin converting enzyme, both related to vascular disorders. Others genes considered are related with voltage dependent channels involved in nociceptive signalling, with neurotransmitters such as dopamine or serotonin and with receptors of the *transient receptor potential* family (TRP) which are expressed in the nociceptive neurons of the trigeminal nerve and whose activation leads to the release of CGRP [16, 18, 32, 33].

There are no many studies considering pharmacogenetics of migraine. Most of them have explored predictors of response of migraine attacks to triptans. So, polymorphisms related to triptan metabolizing enzymes (monoamine oxidase A, CYP3A4, CYP1A2 [34], COMT [35]) to serotonin (5-HT_1B_) and dopamine (DRD2) receptors [36] and to the serotonin transporter gene SLC6A4 [37] have been explored as possible predictors or response to triptans in episodic migraine patients.

Considering preventive therapies, certain mitochondrial DNA haplogroups showed influence in response to riboflavin in migraine patients [38]. Nevertheless, to the best of our knowledge, no previous study has addressed the relationship between the variants and genes included in this study and response to OnabotA.

The relationship between an SNP and a particular phenotype is more consistent when sample size is large, when the gene is located in a related area of genetic linkage (for example in the case of migraine in an area related to pain response) or if the relationship has been demonstrated in animal models [39]. Given the scarcity of individuals suffering from CM who are resistant to preventive oral treatment or OnabotA injection, and especially the low frequency of some haplotypes, the sample size used in this study might be considered as small. Hence, the significant results should be considered with caution even though the same level of significance might be found in other similar groups involved in other independent studies that might validate the findings. It is also important that the subjects being compared here belong to the same ethnic group. In our study, all the patients were of Caucasian ethnicity and of Spanish origin.

Furthermore, the SNPs variant should modify the regulation of the gene (SNP promoter) or its primary structure (SNP exon). Although certain genes can, by themselves, be related to patients having a greater susceptibility to suffer migraine, it sometimes occurs that they are reflecting what is happening in a neighbouring gene, since many alleles segregate themselves in bloc forming haplotypes within which are found several genes. The protein related to such a gene must be implicated in the pathogenesis of the illness. The genes being studied must be selected according to the evidence that there is a probability of association with the illness, on the basis of previous studies which support the relationship between those genes and the pathogenesis of the illness (studies in animal models, in illnesses where there is a familial tendency, etc.) [40]. That was we have tried to do in our design.

The definition of the phenotype of the illness and of its development should be sufficiently homogeneous or serious in patients to make comparison possible. In this case, patients included were diagnosed of CM accordingly to the ICHD-III beta [23] and resistance to previous oral preventatives was clearly defined.

As far as the handling of samples is concerned, it must be meticulous, since a percentage of error in this type of study on the scale of 1–3% has been described, leading to the drawing of false conclusions [41]. To avoid errors from the contamination of samples, sterility measures should be maximised. Also, in the process of genotyping, white control wells and empty control wells were used; repeating the genetic analysis was considered when the degree of allele discrimination was not clear, or not including the results where they were inconclusive as it happened with 7 samples regarding CALCA gen in our study.

Migraine, as other pain disorders, is more prevalent in females. Multiple phenotype differences between male and female migraine have been described. Symptoms as allodynia, response to triptans or preventatives, psychiatric comorbidities, and even brain structures or functional connectivity showed sex differences in migraine [42]. This heterogeneity provides us the opportunity to sub-classify our population and develop a genetic analysis considering only the female sub-population as we have done in this study.

All the variables considered were in Hardy-Weinberg equilibrium except one which deviated from it with a significance of *p* = 0.00006. The SNP in question was rs222749, located on the TRPV1 gene. The lack of equilibrium in the hardy-Weinberg law can be explained either by the small sample size, in which there are very infrequent haplotypes, or by the existence of selection bias. The latter situation is produced when there is a close relationship between the selection criterion, in our case CM, and the SNP. The results relating to whether this variant is a cause of CM are not completely conclusive and so it may be argued that the disequilibrium is due to a very low frequency of one of the alleles (only 4 homozygous patients in our population). However, although there is no literature about the implication of TRPV1 in chronic migraine, this gene has been widely studied like an important molecular player in chronic pain states, like chronic migraine [43].

The SNP rs3781719 c.-767 T > C of the CALCA gene showed significant differences between the group of responders and the group of non-responders to OnabotA. All of the analyses were statistically significant, particularly the hypothesis of dominance of the allele C (*p =* 0.007), whose presence, not only as homozygous but also as heterozygous, impedes the response to OnabotA. The CALCA gene encodes for the peptide CGRP, whose involvement in migraine has been studied widely [44]. CGRP levels have been proposed as a marker of migraine, mainly of chronic migraine [45, 46]. When OnabotA is used in CM patients, a decreasing of interictal levels of CGRP have been shown [47]. Finally, the increased levels of CGRP have been proposed as predictors of efficacy of OnabotA therapy in these patients [19, 48].

Results of our study support the role of CGRP in the therapeutic response of CM and its usefulness as a biomarker or therapeutic target, especially taking into account that the position c.-767 T > C of SNP rs3781719, located in the promoter region 5-UTR, could alter the expression of the gene. Our data suggest a possible regulatory role of the response to OnabotA mediated by the CALCA gene.

The SNP rs222749 located on the gene TRPV1 also showed significant differences between the two groups. Allele A represents 4.17% of the responders, while in the group of non-responders it is 12.5%. This would support the hypothesis that OnabotA interferes with the response mediated by the TRPV1 receptor [14], encoded by that gene, and as a result impedes the release of neuropeptides implicated in pain, such as CGRP or substance P. The reduction of sensitivity to TRPV1 channels could be of interest to explain the effect of OnabotA as migraine preventative [49]. Thus, the patients with allele A in homozygous state would be more susceptible to the release of those neuropeptides.

In our study clinical and demographical variables considered did not correlate with response to OnabotA in CM patients. The series which showed correlation of time from onset of migraine and chronic migraine with good outcome included a quite larger number of patients than our study [20].

We did not consider if the presence of sympathetic or parasympathetic symptoms correlated with the genotype or if other parameters influenced the response. Future studies should explore this possible association in detail.

## Conclusions

In conclusion, in this study, and for the first time, two genetic polymorphisms have been associated with the response to therapy with OnabotA in Chronic Migraine patients. These polymorphisms are situated on the CALCA and TRPV1 genes. However, the role of the SNPs identified has to be considered with caution and the results have to be validated by other independent studies.

## Appendix

**Table 4:** Response to OnabotA

	No (n)	Yes (n)
rs145837941 (CALCA)		
AA	18	63
AG	0	0
GG	0	0
rs1800497 (DRD2)		
AA	1	2
AG	7	25
GG	15	38
rs2201169 (GABRA3)		
TT	28	80
TC	8	38
CC	0	2
rs1139916 (GABRE)		
AA	6	10
CA	14	57
CC	16	53
rs6691840 (GRIK3)		
AA	23	66
AC	13	43
CC	0	9
rs734784 (KCNS1)		
GG	8	29
GA	14	56
AA	13	34
rs1050316 (MEF2D)		
TT	16	51
TG	17	56
GG	3	13
rs121434294 (MTHFR)		
CC	19	68
CT	0	0
TT	0	0
rs2230912 (P2RX7)		
AA	27	85
AG	8	28
GG	1	3
rs7640543 (TGFBR2)		
AA	4	11
AG	17	60
GG	15	49
rs222741 (TRPV1)		
TT	18	64
TC	15	51
CC	2	5
rs222749 (TRPV1)		
AA	3	1
GA	3	8
GG	30	111
rs734312 (WFS1)		
AA	12	34
AG	14	55
GG	9	31
rs3781719 (CALCA)		
TT	9	63
TC	21	45
CC	3	9
rs4354668 (EAAT2)		
GG	3	22
GT	16	54
TT	17	43
rs6627221 (GABRA3)		
CC	1	3
CT	8	44
TT	27	73
rs3810651 (GABRQ)		
TT	7	19
TA	17	55
AA	11	42
rs3813929_1732 (HTR2C)		
TT	1	2
CT	15	35
CC	20	82
rs11172113 (LRP1)		
CC	4	13
CT	18	49
TT	14	58
rs1835740 (MTDH)		
GG	24	73
GA	10	43
AA	2	4
rs1718119 (P2RX7)		
TT	3	17
TC	16	47
CC	17	55
rs6746030 (SCN9A)		
GG	29	88
AG	6	30
AA	1	1
rs10166942 (TRPM8)		
TT	25	85
TC	9	32
CC	2	3
rs222747 (TRPV1)		
GG	21	70
GC	12	43
CC	3	6
rs7217270 (TRPV3)		
AA	9	21
AG	16	54
GG	11	45

**Table 5:** Response to OnabotA. Statistical analysis

FAMILY	GENE	SNP	*p*				
			CHANGE	ANALYSIS BY PHENOTYPE	ALLELE FREQUENCY	ALLELE VARIANT RECESIVITY HYPOTHESIS	ALLELE VARIANT DOMINANCE HYPOTHESIS
Genes related to glutamate homeostasis	MEF2D	rs1050316	G > T	0.907	0.729	0.836	0.665
	LRP1	rs11172113	T > C	0.583	0.439	0.963	0.319
	MTDH	rs1835740	A > G	0.596	0.740	0.527	0.543
	EAAT2	rs4354668	T > G	0.266	0.105	0.147	0.231
	GRIK3	rs6691840	A > C	0.218	0.176	0.088	0.397
CGRP	CALCA	rs3781719	T > C	0.023	0.029	0.794	0.007
		rs145837941	A > G	–	–	–	–
GABA Receptor system	GABRE	rs1139916	A > C	0.312	0.522	0.977	0.148
	GABRQ	rs3810651	A > T	0.824	0.532	0.619	0.604
	GABRA3	rs6627221	T > C	0.271	0.190	0.977	0.074
		rs2201169	T > C	0.381	0.195	0.436	0.205
Voltage dependent channels	SCN9A	rs6746030	G > A	0.400	0.603	0.367	0.419
	KCNS1	rs734784	A > G	0.615	0.458	0.854	0.333
	P2RX7	rs2230912	A > G	0.972	0.863	0.950	0.837
		rs1718119	C > T	0.628	0.584	0.351	0.916
Dopamine receptor	DRD2	rs1800497	C > T	0.775	0.699	0.773	0.569
Serotoninergic receptor	HTR2C	rs3813929	C > T	0.332	0.163	0.675	0.139
TRPfamily	TRPV1	rs222749	G > A	0.040	0.021	0.013	0.102
		rs222747	G > C	0.749	0.740	0.459	0.958
		rs222741	C > T	0.922	0.777	0.843	0.698
	TRPV3	rs7217270	A > G	0.553	0.275	0.446	0.317
	TRPM8	rs10166942	T > C	0.656	0.655	0.361	0.873
Others	WFS1	rs734312	G > A	0.767	0.655	0.498	0.989
	TGFBR2	rs7640543	G > A	0.925	0.920	0.729	0.929
	MTHFR	rs121434294	C > T	–	–	–	–

## Abbreviations

CGRP: Calcitonine gene related peptide
CM: Chronic migraine
DNA: Deoxyribonucleic acid
EDTA: Ethylenediaminetetraacetic acid
GABA: Gamma-aminobutyric acid
GWAS: Genome wide association studies
MTHFR: Methylenetetrahydrofolatereductase
OnabotA: Onabotulinumtoxin A
SNPs: Single nucleotide polymorphisms
TRP: Transient receptor potential action channel

## References

[1] May A, Schulte LH (2016). Chronic migraine: risk factors, mechanisms and treatment. Nat Rev Neurol.

[2] Aurora SK, Dodick DW, Turkel CC, DeGryse RE, Silberstein SD, Lipton RB (2010). OnabotulinumtoxinA for treatment of chronic migraine: results from the double-blind, randomized, placebo-controlled phase of the PREEMPT 1 trial. Cephalalgia.

[3] Diener HC, Dodick DW, Aurora SK, Turkel CC, Degryse RE, Rb L (2010). Onabotulinumtoxin a for treatment of chronic migraine: results from the double blind, randomized, placebo-controlled phase of the PREEMPT 2 trial. Cephalalgia.

[4] Aurora SK, Winner P, Freeman MC, Turkel CC, Re DG, Silberstein SD (2011). OnabotulinumtoxinA form treatment of chronic migraine: pooled analyses of the 56-week PREEMPT clinical program. Headache.

[5] Bendtsen L, Sacco S, Ashina M, Mitsikostas D, Ahmed F, Pozo-Rosich P et al (2018) Guideline on the use of onabotulinumtoxinA in chronic migraine: a consensus statement from the European headache federation. J Headache Pain 19(1) 10.1186/s10194-018-0921-8 PMC6755553 30259200

[6] Matharu M, Pascual J, Nilsson Remahl I, Straube A, Lum A, Davar G (2017). Utilization and safety of onabotulinumtoxinA for the prophylactic treatment of chronic migraine from an observational study in Europe. Cephalalgia.

[7] Pedraza MI, De la Cruz C, Ruiz M, López-Mesonero L, Martínez E, de Lera M (2015). OnabotulinumtoxinA treatment for chronic migraine: experience in 52 patients treated with the PREEMPT paradigm. Springer Plus.

[8] Cernuda-Morollón E, Ramón C, Larrosa D, Alvarez R, Riesco N, Pascual J (2015). Long-term experience with OnabotulinumtoxinA in the treatment of chronic migraine: what happens after one year?. Cephalalgia.

[9] Negro A, Curto M, Lionetto L, Martelletti P (2016) A two years open-label prospective study of OnabotulinumtoxinA 195 U in medication overuse headache: a real-world experience. J Headache Pain 17(1) 10.1186/s10194-016-0591-3 PMC4720620 26792662

[10] Blumenfeld AM, Stark RJ, Freeman MC, Orejudos A, Manack Adams A (2018). Long-term study of the efficacy and safety of OnabotulinumtoxinA for the prevention of chronic migraine. J Headache Pain.

[11] Aicua I, Martínez E, Rojo A, Hernando A, Ruiz M, Carreres A (2016). Real-life data in 115 chronic migraine patients treated with OnabotulinumtoxinA during more than one year. J Headache Pain.

[12] Lucioni A, Bales GT, Lotan TL, McGehee DS, Cook SP, Rapp DE (2008). Botulinum toxin type a inhibits sensory neuropeptide release in rat bladder models of acute injury and chronic inflammation. BJU Int.

[13] Cui M, Khanijou S, Rubino J, Aoki KR (2004). Subcutaneous administration of botulinum toxin a reduces formalin-induced pain. Pain.

[14] Shimizu T, Shibata M, Toriumi H, Iwashita T, Funakubo M, Sato H (2012). Reduction of TRPV1 expression in the trigeminal system by botulinum neurotoxin type-a. Neurobiol Dis.

[15] Aurora SK, Brin MF (2017). Chronic migraine: an update on physiology, imaging, and the mechanism of action of two available pharmacologic therapies. Headache.

[16] Johnson MP, Fernandez F, Colson NJ, Griffiths LR (2007). A pharmacogenomic evaluation of migraine therapy. Expert Opin Pharmacother.

[17] Sutherland HG, Buteri J (2013). Association study of the calcitonin gene-related polypeptide-alpha (CALCA) and the receptor activity modifying 1 (RAMP1) genes with migraine. Gene.

[18] Meents JE, Hoffmann J (2015). Two TRPV1 receptor antagonists are effective in two different experimental models of migraine. J Headache Pain.

[19] Cernuda-Morollón E, Martínez-Camblor P, Ramón C, Larrosa D, Serrano-Pertierra E, Pascual J (2014). CGRP and VIP levels as predictors of efficacy of Onabotulinumtoxin type a in chronic migraine. Headache.

[20] Domínguez C, Pozo-Rosich P, Torres-Ferrús M, Hernández-Beltrán N, Jurado-Cobo C, González-Oria C (2018). OnabotulinumtoxinA in chronic migraine: predictors of response. A prospective multicentre descriptive study. Eur J Neurol.

[21] Hubbard CS, Becerra L, Smith JH, DeLange JM, Smith RM, Black DF (2016). Brain Changes in Responders vs. Non-Responders in Chronic Migraine: Markers of Disease Reversal. Front Hum Neurosci.

[22] Pagola I, Esteve-Belloch P, Palma JA, Luquin MR, Riverol M, Martinez-Vila E (2014). Predictive factors of the response to treatment with Onabotulinumtoxin a in refractory migraine. Rev Neurol.

[23] Headache Classification Committee of the International Headache Society (IHS) (2013). The international classification of headache disorders, 3rd edition (beta version). Cephalalgia.

[24] Chasman DI, Schürks M, Anttila V (2011). Genome-wide association study reveals three susceptibility loci for common migraine in the general population. Nat Genet.

[25] Di Lorenzo C, Grieco GS, Santorelli FM (2012). Migraine headache: a review of the molecular genetics of a common disorder. J Headache Pain.

[26] Freilinger T, Anttila V, De VB, Malik R, Kallela M, Terwindt GM (2012). Genome-wide association analysis identifies susceptibility loci for migraine without aura. Nat Genet.

[27] Schürks M (2012). Genetics of migraine in the age of genome-wide association studies. J Headache Pain.

[28] Carreño O, Corominas R (2012). SNP variants within the vanilloid TRPV1 and TRPV3 receptor genes are associated with migraine in the Spanish population. Am J Med Genet B Neuropsychiatr Genet.

[29] Noch EK, Khalili K (2013). The role of AEG-1/MTDH/LYRIC in the pathogenesis of central nervous system disease. Adv Cancer Res.

[30] Quintas M, Neto JL (2013). Interaction between γ-aminobutyric acid a receptor genes: new evidence in migraine susceptibility. PLoS One.

[31] Ran C, Graae L, Magnusson PK, Pedersen NL (2014). A replication study of GWAS findings in migraine identifies association in a Swedish case-control sample. BMC Med Genet.

[32] Nyholt DR, LaForge KS (2008). A high-density association screen of 155 ion transport genes for involvement with common migraine. Hum Mol Genet.

[33] Ducros A (2013). Genetics of migraine. Rev Neurol (Paris).

[34] Gentile G, Borro M, Simmaco M, Missori S, Lala N, Martelletti P (2011). Gene polymorphisms involved in triptans pharmacokinetics and pharmacodynamics in migraine therapy. Expert Opin Drug MetabToxicol.

[35] Cargnin S, Magnani F, Viana M, Tassorelli C, Mittino D, Cantello R (2013). An opposite-direction modulation of the COMT Val158Met polymorphism on the clinical response to intrathecal morphine and triptans. J Pain.

[36] Ishii M, Sakairi Y, Hara H, Imagawa A, Shimizu S, Takahashi J (2012). Negative predictors of clinical response to triptans in patients with migraine. NeurolSci.

[37] Terrazzino S, Viana M, Floriddia E, Monaco F, Mittino D, Sances G (2010). The serotonin transporter gene polymorphism STin2 VNTR confers an increased risk of inconsistent response to triptans in migraine patients. Eur J Pharmacol.

[38] Di Lorenzo C, Pierelli F, Coppola G, Grieco GS, Rengo C, Ciccolella M (2009). Mitochondrial DNA haplogroups influence the therapeutic response to riboflavin in migraineurs. Neurology.

[39] Colhoun HM, McKeigue PM, Smith GD (2003). Problems of reporting genetic associations with complex outcomes. Lancet.

[40] Hattersley AT, McCarthy MI (2005). What makes a good genetic association study?. Lancet..

[41] Pompanon F, Bonin A, Bellemain E, Taberlet P (2005). Genotyping errors: causes, consequences and solutions. Nat Rev Genet.

[42] Maleki N, Linnman C, Brawn J, Burnstein R, Becerra L, Borsook D (2012). Her versus his migraine: multiple sex differences in brain function and structure. Brain.

[43] Marwaha L, Bansal Y, Singh R, Saroj P, Bhandari R, Kuhad A (2016). TRP channels: potential drug target for neuropathic pain. Inflammopharmacology.

[44] Iyengar S, Ossipov MH, Johnson KW (2017). The role of calcitonin gene-related peptide in peripheral and central pain mechanisms including migraine. Pain.

[45] Cernuda-Morollón E, Larrosa D, Ramón C, Vega J, Martínez-Camblor P, Pascual J (2013). Interictal increase of CGRP levels in peripheral blood as a biomarker for chronic migraine. Neurology.

[46] Riesco N, Cernuda-Morollón E, Pascual J (2017). Neuropeptides as a marker for chronic headache. Curr Pain Headache Rep.

[47] Cernuda-Morollón E, Ramón C, Martínez-Camblor P, Serrano-Pertierra E, Larrosa D, Pascual J (2015). OnabotulinumtoxinA decreases interictal CGRP plasma levels in patients with chronic migraine. Pain.

[48] Domínguez C, Vieites-Prado A, Pérez-Mato M, Sobrino T, Rodríguez-Osorio X, López A (2018). CGRP and PTX3 as predictors of efficacy of OnabotulinumtoxinA type a in chronic migraine: an observational study. Headache.

[49] Zhang X, Strassman AM, Novack V, Brin MF, Burnstein R (2016). Extracranial injections of botulinum neurotoxin type a inhibit intracranial meningeal nociceptors’ responses to stimulation of TRPV1 and TRPA1 channels: are we getting closer to solving this puzzle?. Cephalalgia.

